# Recent Progress in Physiology

**Published:** 1878-06

**Authors:** 


RECENT PROGRESS IN PHYSIOLOGY.—*Air in the Circulatory System.*—The various disturbances which may be produced by the mixture of free air or gas with the circulating blood have been carefully studied by County in several series of experiments on animals which were usually slightly curarized. His conclusions are that bubbles of air mixed with the arterial blood are an obstacle to the flow through the capillaries which may be estimated at several centimeters of mercury, the obstacle increasing with the amount of air. The rapidity with which blood mixed with air circulates through any organ increases, as in the case of normal blood, with a rise of the general blood tension and with a diminution of local vaso-constrictor activity. The obstacle varies very greatly in different organs. Thus "foamy" blood traverses the capillaries of the limbs and brain much more readily than those of the abdominal viscera, a result the opposite from that which might be expected from the relative size of the capillaries in these organs.

The immediate cause of the dangerous and even fatal consequences of mixing air with the blood seems to be a stoppage of the circulation. This stoppage may, according to the author, be brought about in three different ways.

I. By the accumulation of air in the right side of the heart. This accumulation may occur whether the air is introduced by the veins or the arteries, and its effect is in all cases to distend the right side of the heart to two or three times its normal volume, producing mitral insufficiency. The elasticity of the air, which causes it to be compressed instead of driven out by the systole of the right ventricle, and its lightness, which keeps it always in the upper part of the cavities, seem to afford a mechanical explanation of this distension. The distended ventricle contracts less and less completely, and the blood, having a free passage backward through the mitral valve, gradually ceases to circulate.

II. By the direct obstacle to the circulation presented by the air in the capillaries. The stoppage of the circulation thus produced is, of course, more or less localized, and the gravity of the result will depend upon the importance of the organ in which the stoppage occurs. If the lungs are the organs affected, as is commonly the case when air enters the veins, the phenomena of asphyxia will be produced. The paralysis which is so marked a symptom in the so-called "caisson disease" seems to be an affection of this sort, the air set free in the blood by too sudden a "decompression" obstructing the circulation in the nerve centres.

III. By vascular paralysis resulting from long anæmia of the nerve centres. This subject has been studied by the author in a separate series of experiments, in which local anæmia was produced by the injection of lycopodium into the blood-vessels supplying the various nerve centres.

The danger resulting from the entrance of air into the veins has recently been made the subject of a clinical lecture by Fischer, to which valuable exposition the reader is referred for the bibliography of the subject. In experiments on rabbits the author found that a free opening into the internal jugular vein in the lower part of the neck always caused sudden death preceded by an audible sound of the entrance of air and of dyspnœa. The same result generally followed the opening of the axillary vein, but not that of the external jugular or femoral veins. The effect was the same whether the animals were anæmic or full-blooded, starving or well-fed. Autopsies invariably showed the right side of the heart to be distended and filled with foamy blood, which could also be traced into the finest subdivisions of the pulmonary artery. If the air was allowed to enter slowly but constantly, the same fatal result followed, except in those cases in which only small amounts were employed, for example, ten to twenty cubic centimeters in half an hour, an observation quite in accordance with our knowledge of the feeble power of the blood to absorb nitrogen. The author discusses the various views which have been held in regard to the cause of death following penetration of air into the blood-vessels, and, in view of the fact that the heart often continues to beat after the occurrence of the most alarming symptoms, is inclined to adopt the opinion of Magendie and Poiseuille, that asphyxia is, in the majority of cases, the immediate cause of death. In regard to the manner in which asphyxia is brought about, the author adopts the theory of Panum, that the air acts like an embolus in the pulmonary artery and interrupts the normal supply of oxygenized blood to the left ventricle.

*Movements of the Vocal Cords.*—The recent application by Ortel of the so-called stroboscopic method of observing rapid vibrations to the study of the movements of the vocal cords, seems likely to lead to important additions to our knowledge of the physiology of vocalization. This method, which was first employed by Plateau in 1836, consists in observing a body having a rapid but unknown rate of vibration at intervals which can be regulated to correspond to the unknown rate. This object can be accomplished either by illuminating the vibrating body by a rapid series of electrical sparks, or by looking at it through holes in the edge of a revolving disc, or through a perforated disc attached to a tuning-fork. If the rate of the observation coincides with that of the vibrating body, the latter, being observed always in the same phase of its vibration, presents the appearance, owing to the persistence of the impression on the retina, of a body perfectly at rest. If the rate of the observation is a little slower than that of the vibrating body, the latter, being at each observation in a somewhat more advanced phase of its movement, will appear to execute its vibration at a rate corresponding to the difference between the rates of observation and vibration. Thus a body vibrating two hundred times in a second, seen through a stroboscopic apparatus giving two hundred and one observations in a second, will appear to vibrate once in a second. Thus by knowing the rate of the observations and noting the rate of apparent stroboscopic movement, the rate of the vibrating body may be determined. It is also possible by this method to study accurately the appearance presented by a vibrating body in every phase of its movement.

The application of this method to the study of the vocal cords requires the use of the most powerful sources of information; for example, sunlight, electrical light, or lime light. Oertel's observations, which have as yet been given only in preliminary communications, show that in ordinary chest tones the vocal cords vibrate throughout their whole length and breadth in such a way that the excursions of the various points of the cord increase with their distance from the outer border. In falsetto tones, on the other hand, though the cords vibrate in their whole length and breadth, the vibrating surface presents a curved nodal line, about one-third of the distance from the free edge to the attached border, the parts of the cord on opposite sides of the nodal line being of course always in opposite phases of vibration. In very high falsetto tones a second nodal line may be observed, indicating a still further subdivision of the vibrating surface.

Oertel suggests the possibility that this method of research will throw light upon various obscure laryngeal troubles, particularly those affecting singers, dependent, probably upon anomalies of vibration and differences of tension of the vocal cords.

*The Telephone as an Instrument of Research.*—Though so short a time has elapsed since the invention of the telephone, it has already found numerous applications as an instrument of physical and physiological research. Goltz has shown that the electrical currents produced in the conducting wires by the different vocal sounds may be used to irritate the motor nerves of the frog, and that contractions of different intensity correspond to the different vowel sounds, *a* and *o* producing the most powerful, and *i* (as in *mien*) the weakest stimulation. The result is the same whether the conducting wires of the telephone are connected directly with the nerve or with the primary coil of an induction apparatus the secondary currents of which furnish the stimulus.

Du Bois-Reymond has made similar observations, and has also given reasons for regarding the telephonic transmission of sounds as an additional argument in favor of the theory of Helmholtz, that the timbre or "clang-tint" of a sound is not altered by a change in the relative position of the component vibrations, causing them to combine in different phases. For he has shown by a mathematical discussion of the conditions under which the transmission occurs that a change of this sort must take place, and yet the sounds are transmitted unaltered in timbre from one telephone to the other.

Hermann has investigated the subject of telephonic transmission by induction, and has found not only that sounds may be transmitted unaltered when one telephone is connected with the primary and the other with the secondary coil of an induction apparatus (or still better with the two parts of a galvanometer coil), but that the result is not interfered with when several other coils are introduced between the hearing and the speaking telephone, so that the sounds are produced in the latter by induced currents of the third, or even fifth order. In view of this fact, Hermann maintains that tho law of induction, assumed by Du Bois-Reymond in his above-mentioned calculation, cannot, for some reason or other, be applied to oscillatory changes of intensity, such as are produced by the telephone.

Hermann has also employed the telephone as a means of studying the feeble electrical currents of muscles, and finds that when the longitudinal and transverse sections of a muscle are connected by unpolarizable electrodes with a telephone, and a mechanical interrupter is included in the circuit, a noise is heard corresponding to the interruption. That the muscle current is really the cause of the sound is proven by the fact that silence ensues when the electrodes are withdrawn from the muscle and placed in contact with each other. Although the constant current of the muscle at rest is thus readily demonstrated, all attempts to render audible the rapid variations of this current (the "action current" of Hermann) which accompany a tetanic muscular contraction have as yet yielded only negative results. This is the more surprising from the fact that when tested with an ordinary induction apparatus with a vibrating armature and a sliding secondary coil, the telephone responds with an audible sound to induced currents of so feeble intensity that they will not stimulate the most sensitive nerve-muscle preparation; yet when the nerve of this preparation is applied to the surface of a tetanically contracting muscle, the well-known phenomena of secondary tetanus ensues. It seems, therefore, that the telephone is more sensitive than the frog's nerve to the secondary currents of an induction apparatus, while for the electrical currents which accompany muscular activity, the reverse is the case.

To the currents produced by the thermopile, Hermann found the telephone very sensitive. If a telephone, a Heidenhain's thermopile, and a mechanical interrupter are included in an electrical circuit (the latter instrument being placed in an adjoining room on account of the noise produced), it is only necessary to bring a finger into the neighborhood of one of the surfaces of the pile to hear a distinct noise. A similar use of the telephone in connection with a thermopile has also been described by Forbes.

Finally, it should be mentioned that the telephone affords a valuable method of testing the vibrating interrupters for electrical currents so commonly used in physiological researches. It is usually assumed that the number of interruptions correspond to the number of vibrations as determined by the tone of the vibrating spring. The accuracy of this assumption can be readily tested by observing whether a telephone included in the circuit gives the same tone as the interrupter.

It is not yet possible to speak with certainty of the value to the physiologist of that instrument which is so closely allied to the telephone, namely, the phonograph, but it is evident that it cannot fail to render important service in the study of vocalization and articulation. For indications of the problems likely to be solved by its aid, the reader is referred to the communications of Jenkin and Ewing.

